# Large homozygous *RAB3GAP1 gene* microdeletion causes Warburg Micro Syndrome 1

**DOI:** 10.1186/s13023-014-0113-9

**Published:** 2014-10-21

**Authors:** Sylvie Picker-Minh, Andreas Busche, Britta Hartmann, Birgit Spors, Eva Klopocki, Christoph Hübner, Denise Horn, Angela M Kaindl

**Affiliations:** Department of Pediatric Neurology, Charité – Universitätsmedizin Berlin, Campus Virchow-Klinikum, Augustenburger Platz 1, 13353 Berlin, Germany; SPZ Pediatric Neurology, Charité – Universitätsmedizin Berlin, Campus Virchow-Klinikum, Augustenburger Platz 1, 13353 Berlin, Germany; Institute of Neurobiology and Cell Biology, Charité – Universitätsmedizin Berlin, Campus Mitte, Charitéplatz 1, 10115 Berlin, Germany; Institute of Human Genetics, University Medical Center Freiburg, Breisacher Str. 33, 79106 Freiburg, Germany; Department of Pediatric Radiology, Charité – Universitätsmedizin Berlin, Campus Virchow-Klinikum, Augustenburger Platz 1, 13353 Berlin, Germany; Institute of Medical and Human Genetics, Charité – Universitätsmedizin Berlin, Campus Virchow-Klinikum, Augustenburger Platz 1, 13353 Berlin, Germany; Current address: Institute of Human Genetics, University of Würzburg, Biozentrum Am Hubland, 97074 Würzburg, Germany

**Keywords:** RAB3GAP1, WARBM, Warburg micro syndrome, Microcephaly, Intellectual disability, Congenital cataract, Array CGH

## Abstract

Warburg micro syndrome (WARBM) is a genetic heterogeneous disease characterized by microcephaly, intellectual disability, brain, ocular, and endocrine anomalies. WARBM1-4 can be caused by biallelic mutations of the *RAB3GAP1* (RAB3 GTPase-activating protein 1), *RAB3GAP2*, *RAB18* (RAS-associated protein RAB18), or *TBC1D20* (TBC1 domain protein, member 20) gene, respectively. Here, we delineate the so far largest intragenic homozygous *RAB3GAP1* microdeletion. Despite the size of the *RAB3GAP1* gene deletion, the patient phenotype is mainly consistent with that of other WARBM1 patients, supporting strongly the theory that WARBM1 is caused by a loss of RAB3GAP1 function. We further highlight osteopenia as a feature of WARBM1.

**Electronic supplementary material:**

The online version of this article (doi:10.1186/s13023-014-0113-9) contains supplementary material, which is available to authorized users.

## Letter to the editor

**Figure 1:**
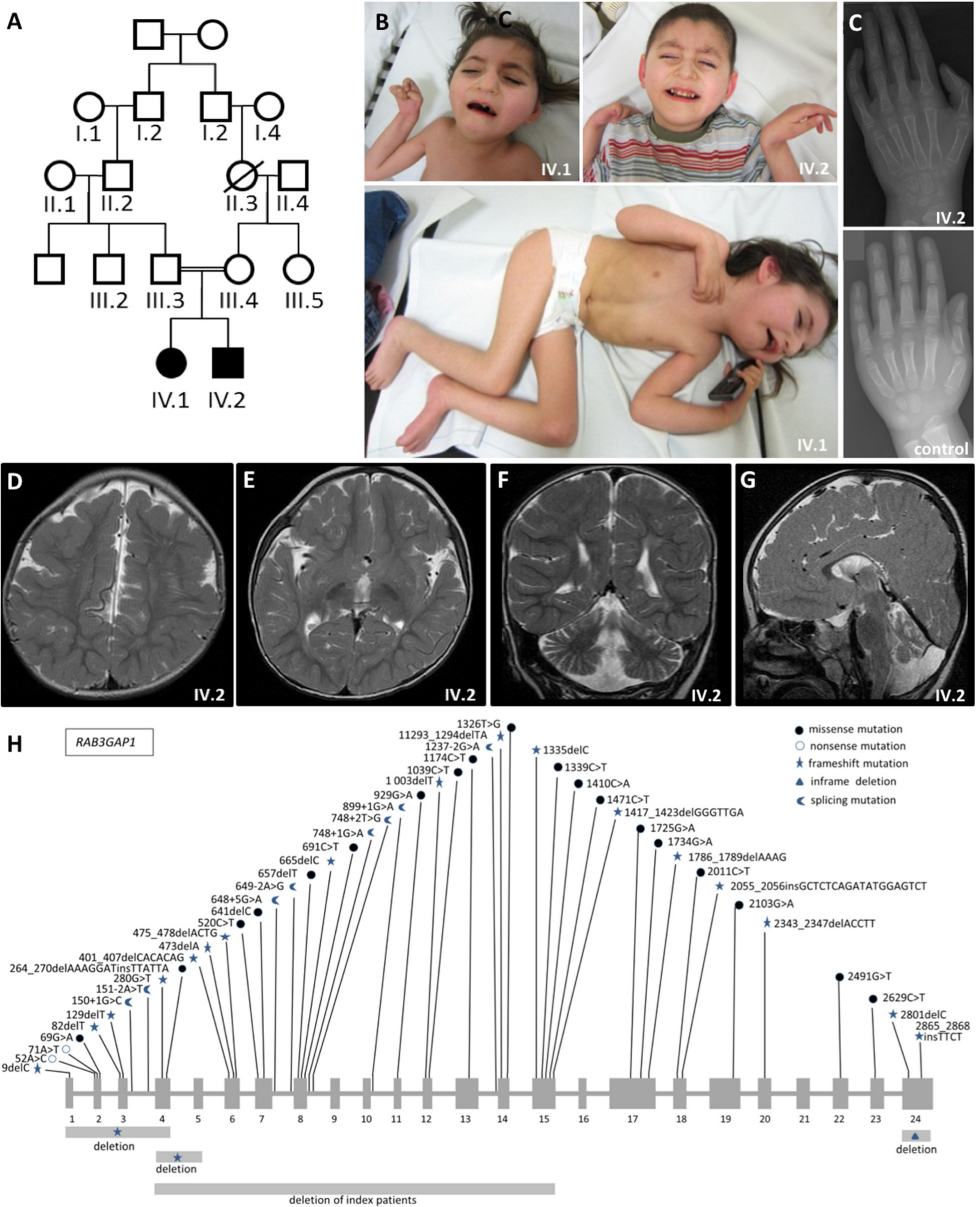
**Phenotype of the index patients with WARBM1. (A)** Pedigree. **(B)** Pictures of the index patients illustrating severe dystrophy, microcephaly, and distal contractures. Facial features include a prominent nasal root, relatively short nose, large ears, and a mild facial hypertrichosis. **(C)** Appropriate skeletal age but severe osteopenia on conventional X-rays of left hand of patient IV.2 when compared to an age- and sex-matched control. **(D-G)** Cranial MRI of patient IV.2 revealed parietal pachygyria (**D**, axial T2), widened sylvian fissure (**E**, axial T2), cerebellar atrophy (**F**, coronal T2), and corpus callosum dysmorphism with agenesis of the splenium corpi (**G**, sagittal T2). **(H)** Scheme depicts all previously reported mutations in the *RAB3GAP1* gene in patients with WARBM1 and the novel deletion in our index patients.

Warburg micro syndrome (WARBM) is a rare autosomal recessive disorder characterized by neurodevelopmental abnormalities such as congenital or postnatal microcephaly, severe intellectual disability, pachy- or polymicrogyria, and hypoplasia/agenesis of the corpus callosum as well as ocular manifestations including congenital cataract, microcornea, microphthalmia, and optic atrophy [1–3]. Further features of WARBM comprise hypothalamic hypogonadism, epilepsy, limb spasticity, and joint contractures. WARBM1 (MIM#600118) is caused by biallelic mutations of the RAB3 GTPase-activating protein 1 gene *RAB3GAP1* (2q31; MIM*602536), WARBM2 (MIM#614225) by mutations of the RAB3 GTPase-activating protein 2 gene *RAB3GAP2* (1q41; MIM*609275), WARBM3 (MIM#614222) by mutations of the RAS-associated protein RAB18 gene *RAB18* (10p12.1; MIM*602207), and WARBM4 (MIM#615663) by mutations in the TBC1 domain protein, member 20, gene *TBC1D20* (MIM*611663). Most mutations were predicted to result in nonsense-mediated mRNA decay and/or loss-of-protein-function [1,2,4–7], putatively explaining the lack of a genotype-phenotype correlation. We here report the largest *RAB3GAP1* gene microdeletion to date in patients with WARBM1 and compare their phenotype with that of other WARBM1 patients. The two index patients were born at term without complications as the first and second child of healthy, consanguineous parents of Kurdish-Armenian descent (Figure 1). Pregnancies were uneventful, and anthropometric data in the first months of life were reported to be normal by the parents. Both patients were diagnosed with bilateral cataracts in the first months of life, and cataract surgery was performed in patient IV.2. The parents noted progressive hypotonia with loss of head control and finally developmental delay when their child did not attempt to roll within the first year of life. At first presentation at 6 (IV.1) and 5 (IV.2) years-of-age, the patients were not able to roll over, sit, stand, or speak, exhibited a short stature, dystrophy, and microcephaly (IV:1: height 90 cm, 16 cm <3. centile, −5.2 SD; weight 11 kg, 4 kg <3. centile, −3.4 SD; head circumference 47 cm, 1.5 cm <3. centile, −2.6 SD; IV.2: height 95 cm, 6 cm <3. centile, −3.3 SD; weight 10,3 kg, 5 kg <3. centile, −3.7 SD; head circumference 45 cm, 4 cm <3. centile, −3.8 SD), and had bilateral cataracts (unilateral iatrogenic aphakia in IV.2), microcornea, and microphthalmia. Bilateral cryptorchidism was present in IV.2. In both patients, poor head control, sparse voluntary movements, axial hypotonia, thoracolumbar scoliosis, lower-limb-spasticity and contractures, and unilateral hip dislocation were apparent. Cranial MRI revealed bilateral parietal pachygyria, dysgenesis of the corpus callosum with agenesis of the splenium, prominent fissura sylvii, mild cerebellar atrophy, and hypotrophic optic chiasma in both patients (Figure 1, Additional file 1: Figure S1). Their short stature was associated with severe osteopenia, mild growth hormone deficiency (levels −2.2 to −3 SDS), but appropriate bone age and normal calcium, phosphate, alkaline phosphatase serum levels (Figure 1). Vitamin D supplementation over 8 months did not improve osteopenia.

We identified the largest intragenic *RAB3GAP1* microdeletion published to date in the index patients through combined Sanger sequencing and array CGH (arr[hg19]2q21.3(135.837.294 × 2,135.857.789 - 135.872.940 × 0,135.896.068 × 2) and arr[hg19]4p16.3(68.185×2,72.477-156.130×1,165.852×2)) and further characterized the deletion breakpoints using multiple PCR amplicons (Figure 1, Additional file 2: Supplemental Data). The additional small 4p16.3-deletion, containing parts of the genes *ZNF718* and *ZNF595* is likely not relevant for the phenotype of the patients. The deletion is very small, encompassing only 84 kb, and overlaps with deletions documented in the normal population in the database of genomic variants (DGV). All deletions listed in decipher affecting the respective chromosome are vastly larger. Moreover, both corresponding genes have not been associated with clinical phenotypes (search in: OMIM, PubMed, Uniprot, Genecards). However, we confirmed a homozygous 2q21.3-deletion of 50.4 kb encompassing exons 4–15 of *RAB3GAP1* corresponding to about 45% of the coding gene sequence: 135,840,320-135,891,847 (hg 19) in both patients, which is heterozygous in the parents as shown by qPCR. The extensive size of the patients´ deletion, their phenotypes resemble previous descriptions of WARBM1 (Table 1), thereby firmly supporting the theory that all WARBM1 phenotypes are caused by a loss of RAB3GAP1 function and/or by nonsense-mediated mRNA decay. Surprisingly, some WARBM1-associated neuro-ophthalmological anomalies were absent in our patients, such as ptosis and nystagmus, delayed myelination, cerebral atrophy, and seizures [1,2,8–11]. Such mild phenotypic variability of WARBM1 is not well understood.

**Table 1:** **Comparison of phenotypic features of the index patients with those described in other patients with Warburg micro syndrome 1–3 and Martsolf syndrome**

**Characteristics and symptoms**	**HPO ID**	**Patient**	**Patient**	**WARBM1**	**WARBM2**	**WARBM3**	**MS**
Pedigree ID		II.1	II.2				
Gender		Female	Male				
Age at last assessment (years)		5.9	4.5				
**Growth**							
Short stature	0004322	+	+	+	+	+	+
Postnatal failure to thrive	0001508	+	+	+	+	+	+
Growth hormone deficiency	0000824	+	+	NR	NR	NR	+
**Head and neck**							
Postnatal microcephaly	0000252	+	+	+	+	+	(+)
Micrognathia	0000347	+	-	+	-	-	+
Large ears	0000400	-	+	+	+	-	-
Microphthalmia	0000568	+	+	+	+	+	+
Microcornea	0000482	+	+	+	+	+	+
Congenital cataract	0000519	+	+	+	+	+	+
Ptosis	0000508	-	-	(+)	-	-	-
Nystagmus	0000639	-	-	(+)	-	-	-
Epicanthal folds	0000286	-	-	-	-	-	+
**Genitourinary**							
Cryptorchism	0086889	NA	+	+	+	+	+
Hypogenitalism	0003241	-	+	+	+	+	+
**Skeletal**							
Osteoporosis	0000939	++	++	NR	NR	NR	NR
Kyphoscoliosis	0002751	+	+	+	NR	+	+
Joint hypermobility	0001382	-	-	(+)	-	-	-
Joint contractures	0002803	+	+	+	+	+	-
Foot deformities	0001760	-	-	+	+	-	+
**Hair**							
Facial hypertrichosis	0002219	+	+	+	-	-	-
**Neurologic**							
Intellectual deficit	0001249	++	++	++	++	++	+
Optic atrophy	0000658	+	+	+	+	+	-
Hyperreflexia	0007034	+	+	+	+	+	+
Muscular hypotonia	0001290	+	+	+	+	+	+
Spastic diplegia	0001264	+	+	+	+	+	(+)
Seizures	0001250	-	-	+	-	+	-
Inability to walk	0002540	+	+	+	+	+	(+)
Absent speech	0001344	+	+	+	(+)	+	(+)
**Cranial MRI**							
Abnormal corpus callosum	0001273	+	+	+	+	+	(+)
Cerebral atrophy	0002059	-	-	+	(+)	+	(+)
Cerebral malformations	0007319	-	-	+	-	-	-
Polymicrogyria	0002126	-	-	+	(+)	+	(+)
Pachygyria	0001302	+	+	+	-	-	-
Enlarged sylvian fissures	0100952	+	+	+	-	+	(+)
Cerebellar hypoplasia	0001321	+	+	+	(+)	+	-
Dysmyelination	0007266	-	-	+	(+)	+	-
**Cardiovascular**							
Cardiomyopathy	0001638	-	-	NR	NR	NR	+
Cardiac failure	0001635	-	-	NR	NR	NR	+
**Respiratory**							
Recurrent infections	0002205	-	-	NR	NR	NR	+

All symptoms are listed according to the nomenclature and the systematics of the OMIM “Clinical Synopsis” and the Human Phenotype Ontology (HPO [14]). Abbreviations: +, present; −, not present; (+) mild or rare; ++, severe; NA, not applicable; NR, not reported; HPO, human phenotype ontology; WARBM1-3, Warburg micro syndrome 1–3; MS, Martsolf syndrome.

Osteopenia present in our patients has not been highlighted in WARBM so far. While osteopenia can result from vitamin D deficiency, it may also be caused by RAB3GAP1 dysfunction itself as RAB3GAP1 arrests the activity of the osteoclastic bone resorption promoter RAB3D [12]. Uncontrolled activity of the latter is associated with bone structure defects in humans [12]. Osteopenia through RAB3GAP1 deficiency is supported by (i) the serum findings in our patients arguing against a severe rachitis secondary to vitamin D deficiency and (ii) the ineffectiveness of vitamin D supplementation with respect to osteopenia in the patients.

In summary, we report that even the largest microdeletion of 45% of *RAB3GAP1* provokes a rather typical WARBM1 phenotype. We thereby strongly support the theory that all truncating *RAB3GAP1* mutations generate a loss-of-protein-function and/or nonsense-mediated-mRNA-decay and therefore result in a similar phenotype. Only hypomorphic *RAB3GAP1* mutations induce the milder Martsolf syndrome phenotype [5,9,13]. Severe osteopenia needs to be considered as a feature of WARBM, and future insight into the role of RAB3D in WARBM may help to understand skeletal abnormalities and assist in establishing a therapeutic approach.

### Consent statement

Written informed consent was obtained from the patients’ legal guardian for publication of this case report and any accompanying images. A copy of the written consent is available for review by the Editor-in-Chief of this journal.

## Additional files

Additional file 1: Figure S1. Cranial MRI of index patient IV.1 with WARBM1. Additional file 2:
**Supplemental Data.**

## Abbreviations

*RAB3GAP1*: RAB3 GTPase-activating protein 1
*RAB3GAP2*: RAB3 GTPase-activating protein 2
*RAB18*: RAS-associated protein RAB18
*TBC1D20*: TBC1 domain protein, member 20
WARBM: Warburg micro syndrome
SDS: Standard deviation score

## References

[1] Aligianis IA, Johnson CA, Gissen P, Chen D, Hampshire D, Hoffmann K, Maina EN, Morgan NV, Tee L, Morton J, Ainsworth JR, Horn D, Rosser E, Cole TRP, Stolte-Dijkstra I, Fieggen K, Clayton-Smith J, Mégarbané A, Shield JP, Newbury-Ecob R, Dobyns WB, Graham JM, Kjaer KW, Warburg M, Bond J, Trembath RC, Harris LW, Takai Y, Mundlos S, Tannahill D, Woods CG, Maher ER (2005). Mutations of the catalytic subunit of RAB3GAP cause Warburg Micro syndrome. Nat Genet.

[2] Morris-Rosendahl DJ, Segel R, Born AP, Conrad C, Loeys B, Brooks SS, Müller L, Zeschnigk C, Botti C, Rabinowitz R, Uyanik G, Crocq M-A, Kraus U, Degen I, Faes F (2010). New RAB3GAP1 mutations in patients with Warburg Micro Syndrome from different ethnic backgrounds and a possible founder effect in the Danish. Eur J Hum Genet EJHG.

[3] Warburg M, Sjö O, Fledelius HC, Pedersen SA (1993). Autosomal recessive microcephaly, microcornea, congenital cataract, mental retardation, optic atrophy, and hypogenitalism. Micro syndrome. Am J Dis Child 1960.

[4] Bem D, Yoshimura S-I, Nunes-Bastos R, Bond FC, Bond FF, Kurian MA, Rahman F, Handley MTW, Hadzhiev Y, Masood I, Straatman-Iwanowska AA, Cullinane AR, McNeill A, Pasha SS, Kirby GA, Foster K, Ahmed Z, Morton JE, Williams D, Graham JM, Dobyns WB, Burglen L, Ainsworth JR, Gissen P, Müller F, Maher ER, Barr FA, Aligianis IA (2011). Loss-of-function mutations in RAB18 cause Warburg micro syndrome. Am J Hum Genet.

[5] Borck G, Wunram H, Steiert A, Volk AE, Körber F, Roters S, Herkenrath P, Wollnik B, Morris-Rosendahl DJ, Kubisch C (2011). A homozygous RAB3GAP2 mutation causes Warburg Micro syndrome. Hum Genet.

[6] Corbeel L, Freson K (2008). Rab proteins and Rab-associated proteins: major actors in the mechanism of protein-trafficking disorders. Eur J Pediatr.

[7] Liegel RP, Handley MT, Ronchetti A, Brown S, Langemeyer L, Linford A, Chang B, Morris-Rosendahl DJ, Carpanini S, Posmyk R, Harthill V, Sheridan E, Abdel-Salam GMH, Terhal PA, Faravelli F, Accorsi P, Giordano L, Pinelli L, Hartmann B, Ebert AD, Barr FA, Aligianis IA, Sidjanin DJ (2013). Loss-of-function mutations in TBC1D20 cause cataracts and male infertility in blind sterile mice and Warburg micro syndrome in humans. Am J Hum Genet.

[8] Graham JM, Hennekam R, Dobyns WB, Roeder E, Busch D (2004). MICRO syndrome: an entity distinct from COFS syndrome. Am J Med Genet A.

[9] Handley MT, Morris-Rosendahl DJ, Brown S, Macdonald F, Hardy C, Bem D, Carpanini SM, Borck G, Martorell L, Izzi C, Faravelli F, Accorsi P, Pinelli L, Basel-Vanagaite L, Peretz G, Abdel-Salam GMH, Zaki MS, Jansen A, Mowat D, Glass I, Stewart H, Mancini G, Lederer D, Roscioli T, Giuliano F, Plomp AS, Rolfs A, Graham JM, Seemanova E, Poo P, García-Cazorla A, Edery P, Jackson IJ, Maher ER, Aligianis IA (2013). Mutation spectrum in RAB3GAP1, RAB3GAP2, and RAB18 and genotype-phenotype correlations in warburg micro syndrome and Martsolf syndrome. Hum Mutat.

[10] Mégarbané A, Choueiri R, Bleik J, Mezzina M, Caillaud C (1999). Microcephaly, microphthalmia, congenital cataract, optic atrophy, short stature, hypotonia, severe psychomotor retardation, and cerebral malformations: a second family with micro syndrome or a new syndrome?. J Med Genet.

[11] Abdel-Salam GMH, Hassan NA, Kayed HF, Aligianis IA (2007). Phenotypic variability in Micro syndrome: report of new cases. Genet Couns Geneva Switz.

[12] Pavlos NJ, Xu J, Riedel D, Yeoh JSG, Teitelbaum SL, Papadimitriou JM, Jahn R, Ross FP, Zheng MH (2005). Rab3D regulates a novel vesicular trafficking pathway that is required for osteoclastic bone resorption. Mol Cell Biol.

[13] Aligianis IA, Morgan NV, Mione M, Johnson CA, Rosser E, Hennekam RC, Adams G, Trembath RC, Pilz DT, Stoodley N, Moore AT, Wilson S, Maher ER (2006). Mutation in Rab3 GTPase-activating protein (RAB3GAP) noncatalytic subunit in a kindred with Martsolf syndrome. Am J Hum Genet.

[14] Robinson PN, Köhler S, Bauer S, Seelow D, Horn D, Mundlos S (2008). The Human Phenotype Ontology: a tool for annotating and analyzing human hereditary disease. Am J Hum Genet.

