# Supplementary figures and images for: Large homozygous RAB3GAP1 gene microdeletion causes Warburg Micro Syndrome 1
Source: Orphanet J Rare Dis. 2014 Oct 21;9:113. doi: 10.1186/s13023-014-0113-9 (PMC4224754; doi:10.1186/s13023-014-0113-9)

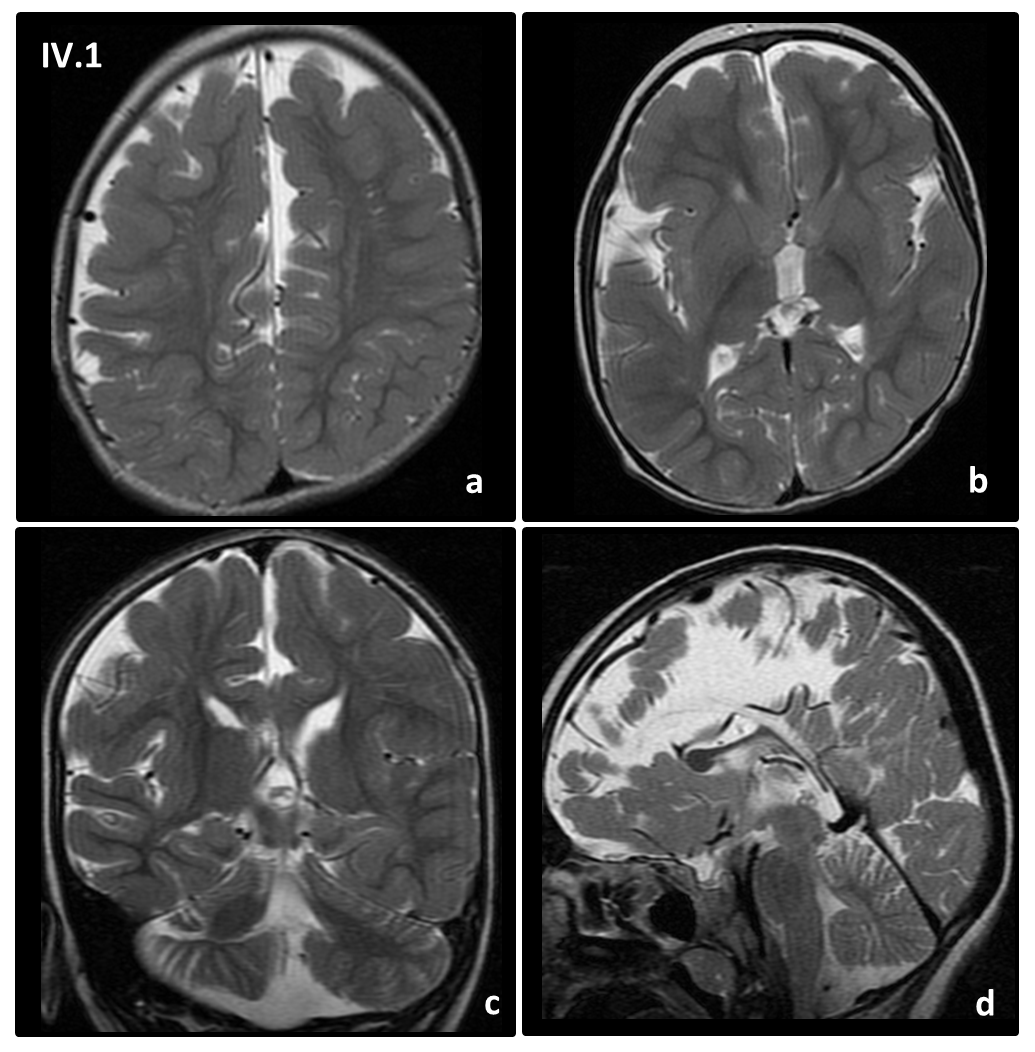

Supplement: Additional file 1: Figure S1. — Cranial MRI of index patient IV.1 with WARBM1. [file 13023_2014_113_MOESM1_ESM.tif]
