# Supplementary material for: Large homozygous RAB3GAP1 gene microdeletion causes Warburg Micro Syndrome 1
Source: Orphanet J Rare Dis. 2014 Oct 21;9:113. doi: 10.1186/s13023-014-0113-9 (PMC4224754; doi:10.1186/s13023-014-0113-9)
Supplement: Additional file 2: — Supplemental Data. [file 13023_2014_113_MOESM2_ESM.doc]

**Supplemental Data**

**Supplemental Figure**

**Figure S1. Cranial MRI of index patient IV.1 with WARBM1.** (A-D) Cranial MRI revealed parietal pachygyria (A, axial T2), widened sylvian fissure (B, axial T2), cerebellar atrophy (C, coronal T2), and corpus callosum dysmorphism with agenesis of the splenium corpi (, sagittal T2).

**Supplemental Material and methods**

Patients. Informed consent was obtained from the parents of the patients for the molecular genetic analysis, the publication of clinical data, radiological imaging data, and photographs. Genomic DNA was extracted from EDTA blood samples using standard procedures. Samples from the patients and her parents were used in this study with approval from the local ethics committees of the Charité (approval no. EA1/212/08 and EA2/163/12).

**Sequencing.** Conventional PCR, quantitative PCR, and Sanger sequencing were performed according to standard procedures established in our laboratory (reference sequence NM_012233.2). Primer sequences are available from the authors upon request.

**Array CGH.** Microarray-based comparative genomic hybridization was performed on Cytochip ISCA 180K array (BlueGnome, Cambridge, UK) with a genome-wide resolution of approximately 70 kb. Data were analyzed using BlueFuse Multi Software (BlueGnome, Cambridge, UK).
